# Influence of Insertion Torque on Clinical and Biological Outcomes before and after Loading of Mandibular Implant-Retained Overdentures in Atrophic Edentulous Mandibles

**DOI:** 10.1155/2019/8132520

**Published:** 2019-06-02

**Authors:** Fernanda Faot, Amália Machado Bielemann, Alessandra Julie Schuster, Raissa Micaella Marcello-Machado, Altair Antoninha Del Bel Cury, Gustavo G. Nascimento, Otacílio Luiz Chagas-Junior

**Affiliations:** ^1^Department of Restorative Dentistry, School of Dentistry, Federal University of Pelotas, RS, Brazil; ^2^Graduate Program in Dentistry, School of Dentistry, Federal University of Pelotas, RS, Brazil; ^3^Department of Prosthodontics and Periodontology, Piracicaba Dental School, State University of Campinas, Piracicaba, SP, Brazil; ^4^Section of Periodontology, Department of Dentistry and Oral Health, Aarhus University, Aarhus, Denmark; ^5^Department of Oral and Maxillofacial Surgery and Maxillofacial Prosthodontics, School of Dentistry, Federal University of Pelotas, RS, Brazil

## Abstract

**Aim:**

To evaluate the influence of primary insertion torque (IT) values of narrow dental implants on the peri-implant health, implant stability, immunoinflammatory responses, bone loss, and success and survival rates.

**Methods:**

Thirty-one edentulous patients received two narrow implants (2.9x10mm, Facility NeoPoros) to retain mandibular overdentures. The implants were categorized in four groups according to their IT: (G1) IT > 10 Ncm; (G2) IT ≥ 10Ncm and ≤ 30 Ncm; (G3) IT >30Ncm and < 45Ncm; (G4) IT ≥ 45Ncm, and all implants were loaded after 3 months of healing. The following clinical outcomes were evaluated 1, 3, 6, and 12 months after implant insertion: (i) peri-implant tissue health (PH), gingival index (GI), plaque index (PI), calculus presence (CP), probing depth (PD), and bleeding on probing (BOP); (ii) implant stability quotient (ISQ) by resonance frequency analysis; and (iii) IL-1*β* and TNF-*α* concentration in the peri-implant crevicular fluid. The marginal bone level (MBL) and changes (MBC) were evaluated. The Chi^2^ test, Kruskal-Wallis test, mixed-effects regression analysis, and the Kendall rank correlation coefficient were used for statistical analysis (*α* = 5%).

**Results:**

G1 presented the highest PD at all evaluated periods. G2 presented higher PI at month 6 and 12. G4 showed increased GI at month 3 and 12 and more CP at month 1 (p=.003). G2 and G4 had higher ISQ values over the study period, while those from G1 and G3 presented lower ISQ values. The IL-1*β* concentration increased until month 12 and was independent of IT and bone type; G4 had a higher IL-1*β* concentration in month 3 than the other groups (p=.015). The TNF-*α* release was negatively correlated with IT, and TNF-*α* release was highest in G1 at month 12. The MBL immediately after surgery and the MBC at month 12 were similar between the groups, and G4 presented a positive MBC at month 12. The survival and success rates were 75% for G1, 81.3% for G2, 64.3% for G3, and 95% for G4.

**Conclusion:**

The IT did not influence the clinical outcomes and the peri-implant immunoinflammatory responses and was weakly correlated with the narrow dental implants primary stability. The observed success rates suggest that the ideal IT for atrophic fully edentulous patients may deviate from the standardized IT of 32 Ncm.

## 1. Introduction

Oral rehabilitation with dental implants aims to establish functional, aesthetic, and phonetic success, with reduced morbidity and pain, aiming at reduced healing and rehabilitation periods with acceptable cost-effectiveness [1]. One of the prerequisites for successful osseointegration of the implants is to achieve adequate primary stability after insertion of the implants to prevent early failures [2]. Many of these failures are biomechanically induced and associated with risk factors such as low primary stability, low bone density, short or narrow implants, and occlusal overloading [3]. Bone density dictates the mechanical properties of the bone bed, which may suffer changes during healing depending on the surgical protocol, since the more porous, more elastic, and well vascularized trabecular bone favors the formation of a dense cortical bone near the surface of the implant, guaranteeing the achievement of biological stability and successful osseointegration [3–5].

Initially, stability of the implant is achieved mechanically and can be measured by the insertion torque of the implant (IT) [6], which is characterized by mechanical bonding between the implant threads and the bone bed and is a measure of the frictional resistance when the implant is inserted into the bone bed [7]. The IT is dependent on bone quality and quantity, surgical technique, and implant geometry [8] and higher IT indicate greater primary stability [5, 9, 10]. The literature indicates that the optimal IT to achieve successful osseointegration is 30 Ncm, which is sufficient to allow both conventional and immediate occlusal loading of the implants while avoiding occlusal overload failures [6, 11].

Although high IT promotes high mechanical primary stability by compacting the host bone [5], it is still discussed whether this stability is advantageous to the bone healing reaction. Implants with high IT in thick cortical bone (Type 1 and Type 2) have been shown to have an increased chance of osseointegration failure, as high IT values can generate an inflammatory reaction of exacerbated healing that contributed to early implant failure [7, 12]. Such defects may have iatrogenic causes arising from overheating by surgical drills, excessive osteoplastic forces, microfractures in the peri-implant bone, debris from the implant surface, reduced vascularization, or local ischemia, which may result in necrosis, increased resorption, and/or bone formation [12–14]. However, a study by Khayat et al. (2013) showed that excessive IT (≥ 70 Ncm) did not affect osseointegration nor increase marginal bone resorption around tapered dental implants, irrespective of the maxillary arch [15]. This contrasts with the results from Marcocini et al. (2018), who investigated 2 types of tapered implants that differed only in the cutting groove design and found that implants installed with high IT (50 Ncm) displayed a larger reduction in mean marginal bone loss values across the follow‐up evaluations up to three years. In addition, this effect was significantly more pronounced in the mandible and the findings also showed that the recession of facial soft tissue was significantly higher in both mandibular and maxillary sites that received high‐IT implants [16].

On the other hand, low IT values are associated with a lower mechanical primary stability due to reduced osseocompression and tension, resulting in a smaller bone-implant contact area. In these cases, the empty space between the implant and the host bone is rapidly filled by the blood clot that will be the precursor of the new bone formation [5, 9]. Thus it is thought that lower stability can promote rapid formation of new bone in the vicinity of the implant without reabsorption of the old bone, promoting rapid secondary stability [17]. The results of a split-mouth, randomized clinical trial by Verrastro-Neto et al. (2018) for the rehabiliatation of the mandible in edentulous patients suggested that the beneficial effect of implants inserted with low IT (19.18±3.56 Ncm) can be observed by the 7th day of healing through high concentrations of biomarkers like vascular endothelial growth factor and osteoprotegerin, which favor angiogenesis and local microcirculation [12, 18].

Norton (2017) [15] performed a study with single implants inserted in both jaws in healed bone beds or immediately after extractions, with a minimum IT of < 5 Ncm (spinners) and maximum IT of 20 Ncm. The results of this study showed that implants installed with IT between 10 and 20 Ncm can reach high success rates comparable to implants installed with high IT, presenting favorable gains in biological stability over time. Implants with ITs greater than 10 Ncm can produce more predictable ISQ values due to less variability in the error bar graph than those with ITs between 5 and 10 Ncm. That is, implants with IT below 10 Ncm present a greater risk of instability even if they have high ISQ, and caution is advised when making decisions regarding occlusal loading of these implants [13].

A systematic review by Berardini et al. (2016) [19] evaluated the effect of high and low IT on marginal bone loss and implant survival in* in vivo* and clinical studies. Their meta-analysis indicated that there were no significant differences in the rate of bone resorption or survival rates between inserted implants with high or low IT in both animal and human studies. However, the authors emphasized that the methodological aspects are described in insufficient detail to allow comparison and that the reported data are heterogeneous. In addition, most available clinical studies describe results from single implant crowns [7, 13–16, 20] or immediate loading protocols adopted in full arch implant restorations [18, 21] and implant-retained overdentures [22–24] or focus on the influence of systemic diseases on IT [25]. There are current no studies investigating the effect of IT in totally edentulous patients with atrophic mandibles.

Therefore, this study investigates whether the peri-implant healing is affected clinically and biologically by IT values, considering that different ITs can generate distinct healing responses. To achieve this, this study monitored the peri-implant health parameters and selected proinflammatory biomarkers (TNF-*α* and IL-1*β*) as secondary variables, along with marginal bone level (MBL) and changes (MBC) over a period of 1 year for narrow diameter implants (NDI) installed as mandibular overdenture (MO)retainers with extremely low (< 10 Ncm), low to moderate (≥ 10 Ncm and ≤ 30 Ncm), moderate to high (>30 Ncm and < 45 Ncm), and high IT values (≥ 45 Ncm). The null hypothesis tested is that the different ITs will not affect the success and survival rates of NDI retaining MO.

## 2. Materials and Methods

This prospective longitudinal clinical trial recruited patients treated at the School of Dentistry of the Federal University of Pelotas, Brazil. The study was approved by the institutional research ethics committee board for human subjects (protocol 1.267.086).

### 2.1. Experimental Design

Between June 2014 and June 2015, wearers of conventional complete dentures (CD) were invited to participate in the study performed at the Federal University of Pelotas - School of Dentistry.

Inclusion criteria:

- ≥ 3 months of adaptation to the conventional complete dentures
- Clinical criteria for mandibular atrophy [26]: poor bone availability in the anterior region of the mandible, poor retention and instability of the mandibular CD
- Availability for follow-up exams at 3, 6, and 12 months
- Signed informed consent form

Exclusion criteria:

- History of radiotherapy in the head or neck region
- Previous history of oral implant treatment
- Treatment with bisphosphonate in the past 12 months
- Heavy smoking (> 11 cigarettes/day)
- Severe diabetes (hyperglycemia or inadequate glycemic control)
- Bleeding disorders (hemorrhagic diathesis; drug-induced anticoagulation)
- Severe systemic diseases (rheumatoid arthritis; osteogenesis imperfecta)
- Compromised immune systems (HIV; immunosuppressive medications) [27]

Individuals who accepted to participate in the study were recruited for treatment with implant-retained mandibular overdentures (IMO). The sample size calculation was based on data from a previous study by Hof et al. (2014) [24] and calculated with the statistical program G*∗*Power ®3.1. The calculation was based on the mean baseline ISQ values of the four IT groups, within an effect size of 1.21, a power of 80%, a 5% alpha error, and an extra increase by 20% to account for potential patient losses and refusals, showing that 15 implants per group were required to complete this study totalizing the need of at least 30 individuals. A total of 40 patients met the inclusion criteria. Of those 31 patients agreed to participate in the study and signed a written informed consent form. Selected patients were evaluated radiographically for bone availability and received 2 narrow diameter implants (NDI) between the mental foramina. After 3 months of healing, Equator type abutments were installed and the mandibular overdentures were loaded.

**Figure 1:**
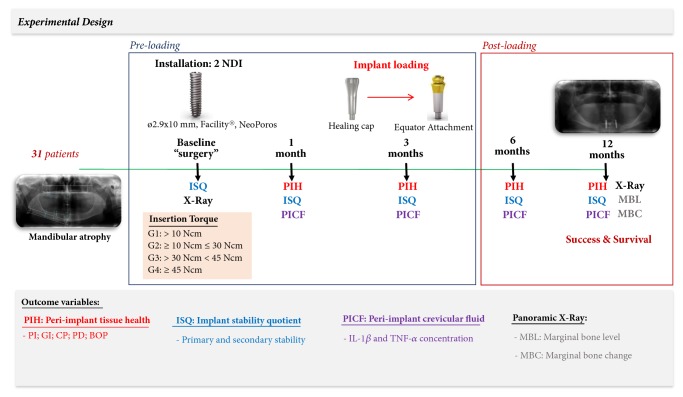
Summary of the experimental design.

The participants were followed and clinically and radiographically evaluated over a period of 12 months. The following peri-implant health parameters were collected over time: plaque index (PI), calculus presence (CP), gingival index (GI), probing depth (PD), and bleeding on probing (BOP). In addition, the implant stability quotient (ISQ) was measured, the peri-implant crevicular fluid (PICF) was collected, and the marginal bone level (MBL) and marginal bone level change (MBC) were measured radiographically. A flow chart summarizing the clinical study is presented in Figure 1.

### 2.2. Radiographic Evaluation and Surgical Protocol

Digital panoramic radiographs were performed to assess the bone availability before surgical planning as previously described [28]. A single expert examiner (R.M.M.M.) performed the linear radiographic measurements to evaluate the mandibular bone height in the anterior and posterior regions. The mandibular atrophy level was then determined following the methodology described by Marcello-Machado et al. (2016) [29].

A standardized one-stage surgical protocol performed by an experienced surgeon (O.L.C.J.) was followed to install two NDI (ø2.9-10mm Facility®, NeoPoros surface, Neodent Osseointegrated Implants, Curitiba, Brazil) in the anterior region of the mandible. The drill was oriented using the distal face of the upper lateral incisors, respecting a distance of 5 mm from the mental foramina. The manufacturer's recommended drill sequence was followed, each implant was inserted at the bone crest level, and the final stage of insertion was performed with a manual wrench. After 3 months of bone healing, the nonsubmerged healing cap was replaced by Equator abutments and the IMOs were loaded by two experienced prosthodontists (A.M.B. and R.M.M.M.). The O-ring attachments that constitute the female part of the Equator abutment were then connected intraorally using self-curing denture acrylic resin (VIPIFlash®, VIPI industry, São Paulo, Brazil) to capture the system to the internal surface of the prosthesis. Denture stability, retention, and occlusion were checked, and the participants received oral hygiene instructions. The radiographic evaluation and surgical protocol used in this study is described in detail by Bielemann et al. (2018) [28]. The implants were categorized into four groups according to the insertion torque (IT) registered during the surgery by a manual wrench: Group 1 (G1), implants with extremely low IT (< 10 Ncm); Group 2 (G2) with low to moderate IT (≥ 10 Ncm and ≤ 30 Ncm); Group 3 (G3) with moderate to high IT (>30 Ncm and < 45 Ncm); and Group 4 (G4) with high IT (≥ 45 Ncm). The implant position was checked by panoramic radiographs performed immediately after installation and analyzed by cross-sectional images obtained with cone bean computed tomography (CBCT) after 1 year of loading to determine the type of cortical bone anchorage (mono- or bicortical anchorage). The anchorage was classified as follows: (i) implants with apical cortical bone contact; (ii) implants with bicortical bone contact (apical and cervical regions); and (iii) implants with cervical cortical bone contact [30].

### 2.3. Implant Stability, Clinical Assessment, Crevicular Fluid Sampling, and Peri-Implant Bone-Level Assessment

A single experienced prosthodontist (A.M.B.) performed all clinical evaluations following the methodology described by Bielemann et al. (2018) [31]. Resonance frequency analysis (RFA) (Osstell®-Integration Diagnostics AB, Göteborg, Sweden), which provides implant stability quotient (ISQ) measurements (scale 1–100), was performed at implant placement (baseline) and after 1, 3, 6, and 12 months. Measurements were made in triplicate in four different directions (mesial, distal, buccal, and lingual).

At 1, 3, 6, and 12 months after implant insertion, the peri-implant health parameters [28] were measured: plaque index (PI) and gingival index (GI) scores, calculus presence (CP), probing depth (PD), and bleeding on probing index (BOP). In addition, the PICF was collected.

Panoramic radiographs with standardized settings were taken immediately after surgery (Baseline) and 12 months after surgery to measure the peri-implant MBL and MBC. The images were analyzed using the DBSWin-VistaScan digital system, and the reference point was the external edge of the implant head during the evaluation of peri-implant bone level [31].

### 2.4. Implant Success and Survival

The success of the implants was evaluated according to the clinical criteria proposed by Misch et al. (2008) [32] and Papaspyridakos et al. (2012) [33]: absence of pain or tenderness upon function, absence of clinical implant mobility, radiographic marginal bone loss <1.5 mm from initial surgery, and absence of infections, dysesthesia, or exudates [32, 33]. Implants that remained* in situ* but did not meet the success criteria were included in the survival group.

### 2.5. Statistical Analysis

The implants were considered as the statistical unit. The Shapiro-Wilk test indicated that all data were non-normally distributed. The chi-square test was used for comparisons between groups of dichotomous variables (PI, CP, GI, BOP, and cortical bone anchorage), and the Kruskal-Wallis test was used for comparisons between continuous variables (PD, ISQ, IL-1*β*, TNF-*α*, MBL, and MBC).

Mixed-effects linear regression analysis was performed to test the effect of follow-up time, IT, bone type, atrophy, and time since edentulism on ISQ, PD, IL-1*β*, and TNF-*α* adjusted for gender, age, smoking status, bleeding on probing, and plaque presence, taking into account individual differences using random intercepts. All variables were standardized to a mean of 0 and a standard deviation of 1 to allow comparison between the estimates; coefficients and respective 95% confidence intervals were then estimated. The first assessed data were used as the reference category for the comparisons.

The Kendall rank correlation coefficient (*R*_*k*_) test was used to verify the relationships between the variables: ISQ, PD, MBL, MBC, IL-1*β*, and TNF-*α*. The results were stratified to be interpreted according the intensity of the correlations as follows: very high, high, moderate, low, and without correlation [34].

Kaplan-Meier survival analysis was used to calculate the survival rate of implants for each group. The level of significance was set at 5%. All statistical analyses were performed using the SPSS Version 22 software (IBM SPSS Statistics 22).

## 3. Results

Table 1 lists the demographic characteristics of the sample population along with the bone remodeling at 12 months, according to the proposed groups in which the implants are the sample unit. A total of 62 implants were installed in the anterior mandible region of 31 totally edentulous patients, 21 females and 10 males, with a mean age of 66.9 years (59–89) years. The mean mandibular time since edentulism was 24.74 ± 13.12 years, the mean bone height in the anterior midline of the mandible was 23.36 ± 3.74 mm, and the superior height from the mental foramen of 4.10 ± 3.40 mm. Group 1 (G1) consisted of 12 implants, Group 2 (G2) consisted of 16 implants, Group 3 (G3) consisted of 14 implants, and Group 4 (G4) consisted of 20 implants. These 4 groups presented similar characteristics in terms of mean age, atrophy (34 implants were inserted in radiographically atrophic mandibles), bone type (38 implants were inserted in bone type II), and proportion of smokers. However, the time since mandibular edentulism was significantly different between the groups, with G2 having the highest mean of 28.38 ±12.45 years (P = 0.018). In the anterior midline of the mandible, the bone height was significantly higher in the G1 (24.43 ± 4.02 mm, P = 0.038). The MBL immediately after insertion of the implants and after 12 months of osseointegration was similar between the groups 0.00 (-1.09–0.78) mm and 0.00 (-1.08–1.06) mm, respectively (P> 0.05). MBC was only positive for G4, 0.29 (-0.77–1.92) mm, but no significant differences were found (P> 0.05). The cross-sectional CBCT images showed that the majority of the implants (n = 28) were inserted with bicortical bone contact (cervical and apical), 24 implants were inserted with cervical bone contact, and 10 implants were inserted with cortical bone contact. The IT was not influenced by the type of implant anchoring (p > 0.05).

Tables S1 and S2 show the comparisons between the medians (min-max) of the implant stability quotient and proinflammatory markers outcomes, respectively, between groups at different evaluation periods.  Table 2 presents the results of the peri-implant health clinical outcomes. The PI, GI, and BOP outcomes were similar between the groups (P > 0.05),while CP was only significantly more prevalent in G4 after 1 month (P = 0.003). In addition, all implants were surrounded by at least 2 mm of keratinized mucosa (data not shown).

**Figure 2:**
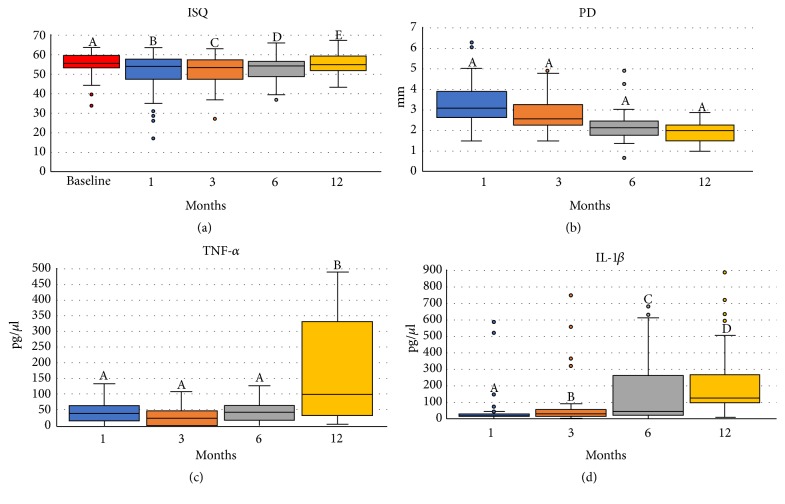
Medians (min-max) of implant stability, probing depth and proinflammatory markers over the evaluation time for al implants (different letters indicate statistically significant differences; the estimates given are standardized coefficients with respective 95% confidence intervals).

**Figure 3:**
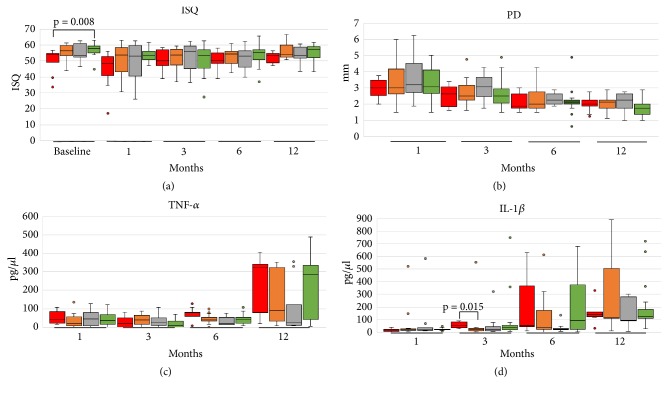
Comparisons between the medians (min-max) of the implant stability, probing depth, and proinflammatory markers outcomes between groups at different evaluation periods (Kruskall-Wallis independent analyses, p<0.05).

**Figure 4:**
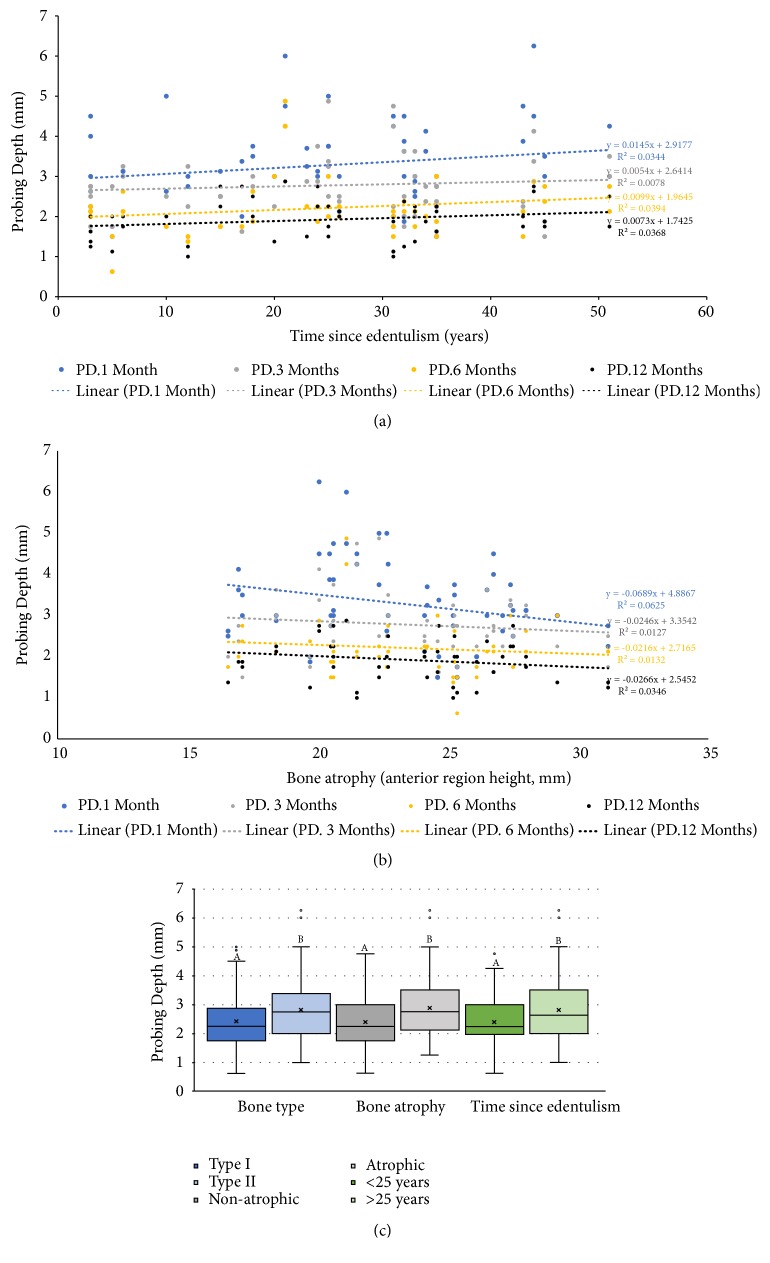
(a) Dispersion diagrams showing the correlations between the probing depth (PD) and time since edentulism and (b) between the PD and bone atrophy according to the anterior mandibular height. (c) Box-plot showing the PD according bone type, bone atrophy, and time since edentulism. Different letters indicate statistically significant differences; the estimates given are standardized coefficients with respective 95% confidence intervals.

Table 3 displays the results from the mixed-effects regression analysis. The ISQ increased gradually over time until 6 months; at 12 months, the ISQ values were similar to those observed at baseline (Figure 2(a)). Implants with low to moderate IT (G2) and high IT (G4) had higher ISQ values over the entire follow-up period, whereas those from G1 and G3 presented lower ISQ values (Figure 3(a) and Table S1). A gradual but nonsignificant reduction in PD values was observed over time (Figure 2(b)). Significantly higher PD values were measured in G3 individuals (Figure 3(b)), implants installed in bone type 2 and in atrophic bone, and individuals with time since edentulism ≥ 25 years (Figure 4(a)). Increased levels of TNF-*α* were observed after 12 months of loading (Figure 2(c)), while reduced values were noted in the medium to high torque (G3) and high torque groups (G4; Figure 3(c)). Finally, IL-1*β* levels tended to increase over the study period, irrespective of torque and bone type (Figures 2(d) and 3(d)).

Table 4 presents the correlation results between bone remodeling, clinical parameters, implant stability, and cytokine concentration. The results indicate a weak positive correlation between IT and primary ISQ (P = 0.01; Rk = 0.252). In G1, a moderate positive correlation was found between MBL and MBC at 12 months and between MBL baseline and IL-1*β* at 6 months. In G2, there was a strong positive correlation between MBL and MBC at 12 months and a moderate positive correlation between the PD in month 1 with MBL at 12 months and between the PD at month 1 and month 3 with the MBC. In G3, a weak positive correlation was found for MBL and MBC at 12 months, along with a moderate positive correlation between MBL baseline and ISQ at 12 months and a weak negative correlation between TNF-*α* and ISQ at month 1. G4 showed a weak to moderate positive correlation between MBL baseline and IL-1*β* at 6 months and a weak negative correlation between MBL baseline and TNF-*α* at month 1.

**Figure 5:**
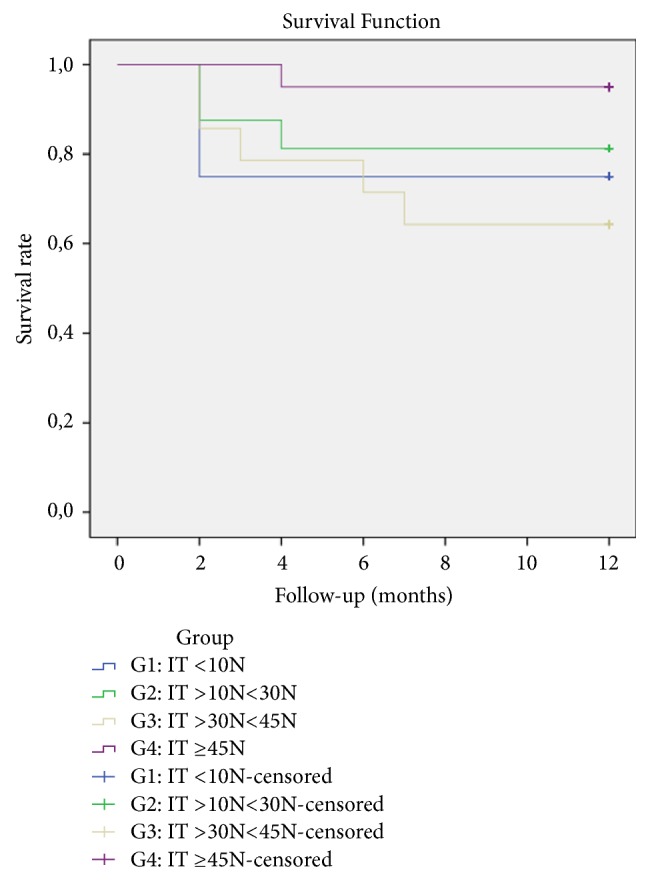
Kaplan-Meier survival curve for each Insertion Torque group.

Figure 5 shows the Kaplan-Meier survival curve of the implants. In the G1 group, 3 implants failed at 2 months resulting in success and survival rates of 75%. The success and survival rates in G2 were 81.3%, as 2 failures occurred before occlusal loading at 3 months and 1 failure occurred at 4 months. G3 had the worst success and survival rates of 64.3%, as 3 implants failed before 3 months and 2 more failures occurred at 6 and 7 months, respectively. G4 had the highest survival and success rates of 95%, with one failure at 4 months.

## 4. Discussion

The influence of IT on outcomes related to implant healing and stability during and after implant osseointegration is still controversial and dependent on factors such as bone availability, patient profile, surgical protocol, and implant macrogeometry [21, 24, 35–37]. Thus, this longitudinal clinical study evaluated the effect of IT values on implant success and survival during 1 year of function and mapped clinical and biological endpoints of NDI placed in the anterior region of totally edentulous jaws. In the studied population, the null hypothesis tested was rejected because the different ITs had different survival and success rates. The high IT group (G4) had the best survival and success rates of 95% while the medium to high IT group (G3) had the worst rates of 64.3%. Multilevel regression analysis enabled evaluating how time, IT, bone type, atrophy, and time since edentulism influence ISQ, PD, and the proinflammatory markers IL-1*β* and TNF-*α*.

This study showed that the PD reduced significantly over the 12 months, independent of the IT. The ISQ decreased from the baseline to 1 month and increased significantly by month 12. In the high torque G4 group, the ISQ showed a smaller reduction compared to the baseline, reflecting the relatively small changes in ISQ when the baseline value is already high. The release of the proinflammatory cytokine IL-1*β* was not influenced by IT, while TNF-*α* was negatively correlated with IT. Factors inherent to the patient such as bone type, atrophy, and time since mandibular edentulism were shown to influence only the PD outcome (Figures 4(a), 4(b) and 4(c)). The IT was not influenced by the bone type nor by atrophic jaw conditions. However, some patient characteristics were significantly different in 2 groups: (i) time since edentulism in the mandible was significantly longer in the low to moderate insertion torque group (G2: 28.38 ± 12.45 years, P = 0.018); and (ii) bone availability in the anterior mandible was significantly higher in the low insertion torque group (G1: 24.43 ± 4.02 mm, P = 0.038).

The peri-implant health indexes show similar behavior in all groups over the follow-up period. The PI was relatively high in the study population, which has a high average age and prolonged period of mandibular time since edentulism. Previous studies have shown that patients with such characteristics may present motor difficulties that inhibit adequate hygienic maintenance of dental implants [28, 31]. The G4 group showed the highest IT and presented higher inflammatory indexes (GI and BOP) during pre-loading and at 6 months after loading. The behavior of these outcomes could be related to maladaptation to the prostheses or reduction of peri-implant mucosal height resulting in exposure of the transmucosal part of the prosthetic abutment, favoring plaque accumulation. It is also recommended re-evaluate the transmucosal height of the prosthetic abutments and its necessity of replacement to promote better adaptation and stability of the mandibular prostheses and maintenance of ideal biological sealing [31]. Thus, periodic consultations are recommended during the first year of implant healing and adaptation, mainly in patients with prolonged time since edentulism using IMO [31].

Although IT did not influence the PD during the 12 months of follow-up, at month 12, PD was reduced by 140% in relation to month 1. This result was expected due to the peri-implant soft tissue healing that increases the bundles of collagen fibers parallel to the surface of the implant and promotes tissue resistance around the prosthetic components [31, 38]. In addition, the PD decreased with shorter time since edentulism (Figure 4(a)) and greater bone availability in the anterior region of the mandible (Figure 4(b)). This corroborated the study by Ivanovski & Lee (2018), who affirmed that the peri-implant mucosa width is genetically predetermined and that the dimensions of this soft tissue are also preserved through the more apical establishment of the implant; that is, marginal bone height determines the biological width of peri-implant soft tissue [38]. The G4 group with the highest IT had a mean PD that was 30% lower than the other groups. This is also consistent with the findings reported by Marconcini et al. (2018), which showed that higher insertion torque (≥ 50 Ncm) in mandible led to greater bone resorption and mucosal recession than that registered for implants placed with a regular IT (< 50 Ncm). Moreover, sites with a thick buccal bone wall (≥ 1 mm) showed smaller recession at the facial soft tissue level only after 3 years [16].

Recently, studies have stated that there is no correlation between IT and the primary stability registered by ISQ [13, 21, 30, 39], indicating that both methods are not comparable [39]. This study found a weak correlation between IT and primary ISQ (P = 0.01; Rk = 0.252), which has previously been reported [39–41]. The present study also showed that there was a drop in ISQ values after primary stability establishment up to 6 months that was subsequently counteracted by the increase in secondary ISQ recording to baseline ISQ values (Table 3 and Figure 2), in accordance with most previously published results [13, 21, 24, 37]. The ISQ of G2 and G4 was approximately 7.5 times higher than G1 and 3 times higher than that of G3 (Table 3 and Figure 3). These results show that the G2 and G4 ITs generate more effective results to achieve an adequate secondary stability, reinforced by the increase in ISQ in these groups after loading up to the 12th month, since G1 and G3 had a reduction in ISQ between 3 and 6 months, followed by an increase between 6 and 12 months. Similar behavior has been reported by Norton (2017), who observed increased ISQ after the third month of osseointegration and occlusal loading of implants inserted with (extremely) low IT from < 5–20 Ncm [13]. Our results are in agreement with Marcello-Machado et al. (2018), where NDI as IMO retainers reached ISQ values similar to those during installation at 12 months of osseointegration, thus demonstrating adequate secondary stability establishment [31]. In our study it was also noted that the G4 ISQ reduced 80% less than the G1, evidencing that minor changes in the ISQ are expected when the value recorded in the baseline is already high [13], and the reduction is more pronounced between baseline and month 1. The proinflammatory markers IL-1*β* and TNF-*α* may enable osteoclastogenesis and reabsorption of alveolar bone, especially during the initial bone healing period [12]. Our results showed that the release of these inflammatory markers had no correlation with each other. However, the 2 cytokines monitored in our study were differently influenced by the IT (Table 3 and Figure 3). The concentration of TNF-*α* showed a significant increase by 110% at 12 months compared to the thrid month, as already observed at 12 months in a study with IMO retained by a bar-clip [42]. Thus, it is suggested that the occlusal loading after 3 months of healing stimulates the release of proinflammatory factors, i.e., the micromovements generated by the loading may have a positive effect on bone neoformation [43], favoring remodeling and bone formation [12, 44]. This effect may be more noticeable when an inadequately low IT is achieved, as evident in G1, which presented a TNF-*α* concentration that was 40% and 30% higher than G2 and G4, respectively, suggesting that the instability causes a more exacerbated TNF-*α* proinflammatory response.

After the first month of bone healing, which is characterized by intense cellular and proinflammatory activity, the TNF-*α* release reduced for all groups until the third month, as expected [12], and this reduction was more pronounced in the high torque group G4. Similar behavior has been demonstrated in the same period (1 and 3 months) in MO-retaining implants with IT > 30 Ncm that received immediate loading. Conversely, G1 and G4 had a mean TNF-*α* concentration that was 80% higher than the G3 and G2 groups at month 12. This high concentration of G4 corroborates the results of the study with an immediate loading protocol by Verrastro-Neto et al. (2018), which suggests that the high IT favors bone formation and repair, as evidenced by the greater osteoblastic activity due to the increase of BMP-9, supraregulation of periostin, involved in the recruitment of osteoblastic cells, and increased levels of placental growth factor, which is involved in bone formation and repair [18].

IL-1*β* has been associated as a marker of bone resorption, peri-implant infection, trauma, and iatrogenic conditions [45–47]. However, in this study, IL-1*β* was not able to reflect the trauma generated during surgery as previous reported by Hof et al. (2014) [24]. Independently of the IT values achieved, as the concentration of IL-1*β* increased progressively over time, with a more expressive increase after occlusal loading, finally it peaked at 12 months with concentrations 140% higher than at first month. Thus, the progressive release of this biomarker may have resulted from microtrauma caused by the functional loading of the implants but imperceptible by the patient stimulating the interaction between the biological events and the mechanical forces, which are fundamental for treatment success. These results are consistent with the results from Elsyad et al. (2017), who attributed this finding to the presence of plaque and gingival inflammation suggesting that IL-1*β* is present in PICF irrespective of the presence of gingival bleeding [48]. In the follow-up study by Hof et al. (2014) that evaluated the impact of low IT (20 Ncm) and high IT (>50 Ncm) during preloading (3 months) and postloading (12 months), it was reported a higher concentration of IL-1*β* in the low torque group; however this finding was not significant. Their IL-1*β* results demonstrated no active stages of tissue destruction, as the concentrations were comparable to those reported for peri-implant health sites [24]. A study that followed implants with a IT of 30 Ncm with immediate loading found that the concentration of IL-1*β* was initially low and it increased progressively until 12 months [48]. Thus, it is suggested that the functional loading of the implants favors IL-1*β* release, interacting with the processes of bone remodeling and osseointegration [12, 37, 44].

Finally, the groups studied did not present similar success and survival rates, G3 with intermediate to high torque had the worst rates (64.3%) as 5 implants failed, and G4 had the best rates (95%) with only one failure after occlusal loading. Moreover, G4 also had the highest ISQ across the different time intervals, with the lowest stability reduction relative to the other groups, higher IL-1*β* concentration, and lower TNF-*α* concentration at month 12, positive bone remodeling in addition to a positive correlation between MBL baseline and the ISQ 12 months (P = 0.033; Rk = 0.618). These results are in agreement with the* in vivo* study of Rea et al. (2015), wherein higher IT showed a tendency to exhibit lower bone crest resorption but also had a reduced implant bone contact [17]. However, another clinical study found that single implants installed with an IT of 68.3 (6.0) Ncm had a 32-fold greater bone loss rate than regular IT implants installed with 30.4(±6.1) Ncm, within 3 months of healing. After occlusal loading at 12 months, this rate decreased drastically, but was still 2 times higher in the high IT group [20]. Thus, elevated IT can compromise marginal bone remodeling due to osseocompression in mandibular cortical bones [16]. Although one recent study found that bicortical bone contact increases the implant stability [30], the type of anchorage did not significantly influence the IT in this study (p > 0.05).

The limitations of this study include the use of NDI without using other types of implants as a control group; the IT was determined with a manual wrench and the sample was split in 4 relatively small IT groups to provide results on the influence of IT during osseointegration, both pre- and postoperative occlusal loading. However, in the correlation analysis, all IT data are grouped together.

## 5. Conclusion

The null hypothesis was rejected because IT was associated with different success and survival rates, although IT did not significantly influence clinical peri-implant health outcomes nor IL-1*β* or TNF-*α* biomarker expression. The survival and success rates suggest that the ideal IT for atrophic fully edentulous patients may deviate from the standardized IT of 32 Ncm. In this study, implants with IT > 45 Ncm presented more favorable clinical and biological results after 12 months of osseointegration. Weak correlations between IT and primary stability were observed in this study; the results also demonstrate that the expected probing depth is a function of time since edentulism, bone type, and mandibular atrophy.

## Figures and Tables

**Table 1:** Patients characteristics according to the insertion torque groups.

	G1 >10Ncm (n=12)	G2 ≥10Ncm ≤30Ncm (n=16)	G3 >30Ncm <45Ncm (n=14)	G4 ≥45Ncm (n=20)
Gender (Female/Male)	9//3	14//2	8//6	11//9
Age (years; mean, SD)	65.83(9.73)	70.19(7.46)	63.65(6.66)	63.65 (6.66)
Mandibular Time since edentulism (years; mean, SD)	22.17(9.88)	28.38(12.45)	22.1(15.98)	22.1(15.98)
Mandibular Time since edentulism (<25 / >25 years)	7/5	3/13	7/7	7/13
Mandibular anterior midline (mm; mean, SD)	24.43(4.02)	23.64(3.01)	22.93(4.56)	22.92(4.56)
Superior bone height from the mental foramen (mm; mean, SD)	3.60(2.65)	3.66(2.87)	3.38(3.63)	3.38(3.63)
Smokers / Non-Smokers	1//11	3//13	1//13	3//17
Bone Atrophy (Yes/ No)^+^	7//5	6//10	9//5	12//8
Bone Type (Type I / Type II)*∗*	2/10	6/10	4/10	12/8
MBL_ baseline (median; min – max)	0.00 (-0.82 – 0.71)	0 (-0.37 – 0.78)	0 (-0.78 – 0.0)	-0.23 (-1.09 – 0.77)
MBL_1 year (median; min – max)	0.00 (-0.36 – 0.83)	0.00 (-1.08 – 0.55)	0 (-0.97 - 0.66)	0 (-0.75 -1.06)
BLC (median; min – max)	0.00 (-1.01 – 0.83)	0 (-1.08 - 0.91)	0 (-0.51 – 1.44)	0.29 (-0.77 – 1.92)
Cortical Bone Anchorage (apical/ bicortical / cervical)	3/2/7	2/7/7	0/8/6	5/11/4

**Table 2:** Presence of peri-implant health issues across the follow-up period (in %) and the significant intergroup comparisons (Chi-square test, p<0.05). *∗* p=0.003.

	Plaque Index				Calculus Presence				Gingival inflammation				Bleeding on Probing			
	1M	3M	6M	12M	1M	3M	6M	12M	1M	3M	6M	12M	1M	3M	6M	12M
G1	50	50	44.4	11.1	0	0	11.1	0	0	0	0	0	0	0	0	0
	6/12	5/10	4/9	1/9	0/12 A	0/10	9/9	9/9	0/12	0/10	0/9	0/9	0/12	0/10	0/10	0/9
G2	25	57.1	61.5	46.2	0	0	0	0	0	7.1	0	0	12.5	7.1	0	7.7
	4/16	8/14	8/13	6/13	0/16 A	0/14	0/13	0/13	0/16	1/14	0/13	0/13	2/16	1/14	0/13	1/13
G3	35.7	58.3	22.2	0	0	0	0	0	0	0	0	0	0	0	11.1	0
	5/14	7/12	2/9	0/9	0/14 A	0/12	0/9	0/9	0/14	0/14	0/9	0/9	0/14	0/12	1/9	0/9
G4	60	55	36.8	31.6	30	0	0	0	0	10	0	5.3	0	5	0	5.3
	12/20	11/20	7/19	6/19	6 /20 B*∗*	0/20	0/19	0/19	0/20	2/20	0/19	1/19	0/20	1/20	0/19	1/19

**Table 3:** Results from the mixed-effects multilevel analysis of the effects time and insertion torque on the clinical and biological conditions related to implant healing. The estimates are given as standardized coefficients with their respective 95% confidence intervals. Analyses were adjusted for gender, age, smoking status, bleeding on probing, and plaque. Statistically significant results are presented in italic.

	ISQ	PD	TNF-*α*	IL-1*β*
	Coef. (95%CI)	Coef. (95%CI)	Coef. (95%CI)	Coef. (95%CI)
*Time*				
Baseline	Ref.	-	-	-
1 Month	*-0.7 (-1.0;-0.4)*	Ref.	Ref.	Ref.
3 Months	*-0.6 (-0.9;-0.4)*	*-0.5 (-0.7;-0.3)*	-0.2 (-0.6;0.2)	*0.4 (0.1;0.6)*
6 Months	*-0.6 (-0.8;-0.3)*	*-1.1 (-1.4;-0.8)*	0.1 (-0.1;0.4)	*0.8 (0.5;1.2)*
12 Months	-0.2 (-0.5;0.0)	*-1.4 (-1.7;-1.1)*	*0.9 (0.5;1.3)*	*1.4 (1.1;1.7)*

*Torque type*				
G1	Ref.	Ref.	Ref.	Ref.
G2	*0.7 (0.1;1.2)*	0.3 (-0.1;0.7)	-0.3 (-0.6;0.1)	-0.1 (-0.5;0.2)
G3	0.4 (-0.3;1.1)	*0.4 (0.0;0.8)*	*-0.4 (-0.8;0.0)*	-0.2 (-0.6;0.1)
G4	*0.8 (0.2;1.3)*	0.1 (-0.3;0.4)	*-0.3 (-0.6;0.0)*	0.0 (-0.3;0.3)

*Bone type*				
1	Ref.	Ref.	Ref.	Ref.
2	0.0 (-0.4;0.4)	*0.4 (0.1;0.7)*	0.1 (-0.2;0.3)	0.0 (-0.3;0.2)

*Atrophy*				
No	Ref.	Ref.	Ref.	Ref.
Yes	0.0 (-0.4;0.4)	*0.3 (0.1;0.6)*	-0.1 (-0.3;0.2)	-0.1 (-0.4;0.3)

*Time of edentulism*				
< 25 years	Ref.	Ref.	Ref.	Ref.
≥ 25 years	-0.1 (-0.5;0.3)	*0.3 (0.1;0.6)*	-0.1 (-0.4;0.1)	0.1 (-0.2;0.3)

**Table 4:** Correlation between implant stability quotient, cytokine concentrations (pg/*μ*l), clinical parameters, and marginal bone relation at each evaluation period. *R*_*p*_ values are Pearson correlation coefficients, and *R*_*s*_ values are Spearman correlation coefficients.

	BASELINE	1 MONTH	3 MONTHS	6 MONTHS	12 MONTHS

	MBL.0				MBC

G1_MBL.12M					p= 0.023 *R*_*k*_ =0.636
G2_MBL.12M					p≤0.001 *R*_*k*_ =0.867
G3_ SUP.HF	p= 0.010 *R*_*k*_ =-0.582				p= 0.020 *R*_*k*_ =-0.612
G3_ ANT.MH	p= 0.015 *R*_*k*_ =0.550				p= 0.030 *R*_*k*_ =0.580
G3_MBL.12M					p= 0.002 *R*_*k*_ =0.853

	IPS	IPS	IPS	IPS	IPS

G2_MBL.12M		p= 0.026 *R*_*k*_ =0.508			
G2_MBC		p= 0.003 *R*_*k*_ =0.662	p= 0.016 *R*_*k*_ =0.539		
G3_MBL.0		p= 0.045 *R*_*k* *t*_=0.332		p= 0.008 *R*_*k*_ =0.476	
G4_MBC		p= 0.030 *R*_*k*_ =-0.366			

	ISQ	ISQ	ISQ	ISQ	ISQ

G3_TNF- *α*.1M		p= 0.024 *R*_*k*_= -0.456			
G3_MBL.0					p= 0.033 *R*_*k*_ = 0.618

	TNF-*α*	TNF-*α*	TNF-*α*	TNF-*α*	TNF-*α*

G4_MBL.0		p= 0.018 *R*_*k*_ =-0.401			

	IL-1*β*	IL-1*β*	IL-1*β*	IL-1*β*	IL-1*β*

G1_MBL.0				p= 0.007 *R*_*k*_ =0.629	
G4_MBL.0				p= 0.037 *R*_*k*_=0.356	
G4_MBL.12m		p= 0.005 *R*_*k*_ =0.461			p= 0.026 *R*_*k*_=0.396
G4_PD12m					p= 0.041 *R*_*k*_ =-0.358

SUP.HF, superior height of the formanina; ANT.MH, anterior midline height; MBL, manrginal bone level; MBC, marginal bone level change; PD, probing depth.

## Data Availability

The data used to support the findings of this study are available from the corresponding author upon request.

## Supplementary Materials

Supplementary Materials Tables S1 and S2 list medians (min-max) and present the comparisons between groups at different evaluation periods for the implant stability quotient and proinflammatory markers, respectively.
