# Supplementary material for: Influence of Insertion Torque on Clinical and Biological Outcomes before and after Loading of Mandibular Implant-Retained Overdentures in Atrophic Edentulous Mandibles
Source: Biomed Res Int. 2019 Jun 2;2019:8132520. doi: 10.1155/2019/8132520 (PMC6582836; doi:10.1155/2019/8132520)
Supplement: Supplementary Materials — Tables S1 and S2 list medians (min-max) and present the comparisons between groups at different evaluation periods for the implant stability quotient and proinflammatory markers, respectively. [file 8132520.f1.docx]

Faot F, Bielemann AM, Schuster AJ, Marcello-Machado RM, Del Bel Cury AA, Nascimento GG, Chagas-Junior OL. *“Influence of insertion torque on clinical and biological outcomes before and after loading of mandibular implant-retained overdentures in atrophic edentulous mandibles”* Biomed Research International 2019

**Table S1:** Comparisons between the medians (min-max) of the implant stability quotient between groups (Kruskall-Wallis independent analyses, p<0.05), at different evaluation periods.

|  | **ISQ** | | | | |
| --- | --- | --- | --- | --- | --- |
|  | **BASELINE** | **1M** | **3M** | **6M** | **12M** |
| **G1** | 54 | 48.5 | 50.25 | 50.5 | 53.5 |
|  | 33.5 - 56.75 | 17.0 - 56.0 | 39.0 - 58.5 | 39.0 - 58.0 | 47.0 - 56.5 |
|  | A | A | A | A | A |
| **G2** | 56.5 | 53.75 | 53.875 | 54.5 | 54 |
|  | 44.0 - 61.25 | 30.75 - 63.0 | 37.0 - 59.5 | 42.5 - 61.0 | 50.75 - 66.75 |
|  | AB | A | A | A | A |
| **G3** | 53.25 | 53 | 56 | 53 | 53.25 |
|  | 46.5 - 62.5 | 26.0 - 62.75 | 36.5 - 62.25 | 40.0 - 62.25 | 43.5 - 60.75 |
|  | AB | A | A | A | A |
| **G4** | 57.75 | 53.5 | 53.25 | 55.25 | 57.25 |
|  | 45.0 - 63.0 | 47.25 - 61.5 | 27.25 - 62.5 | 37.0 - 65.5 | 43.25 - 61.75 |
|  | B | AB | AB | AB | AB |

The letters show the differences between the intergroup comparisons.

Faot F, Bielemann AM, Schuster AJ , Marcello-Machado RM, Del Bel Cury AA, Nascimento GG, Chagas-Junior OL. *“Influence of insertion torque on clinical and biological outcomes before and after loading of mandibular implant-retained overdentures in atrophic edentulous mandibles”* Biomed Research International 2019

**Table S2:** Comparisons between the medians (min-max) of the pro-inflammatory markers between groups (Kruskall-Wallis independent analyses, p<0.05), at different evaluation periods.

|  | **TNF-α** | | | | **IL-1β** | | | |
| --- | --- | --- | --- | --- | --- | --- | --- | --- |
|  | **1M** | **3M** | **6M** | **12M** | **4** | **3M** | **6M** | **12M** |
| **G1** | 41.54 | 18.97 | 77.53 | 324.20 | 13.71 | 58.19 | 52.45 | 139.23 |
|  | 16.13 - 107.99 | 0 - 80.81 | 8.15 - 128.01 | 20.38 - 407.15 | 0 - 42.82 | 28.9 - 89.68 | 11.0 - 629.01 | 31.01 - 343.99 |
|  | A | A | A | A | A | A | A | A |
| **G2** | 18.34 | 38.34 | 39.62 | 90.28 | 23.60 | 23.75 | 35.59 | 119.14 |
|  | 0 - 134.48 | 0 - 87.27 | 0 - 98.0 | 8.9 - 350.84 | 2.78 - 522.84 | 3.57 - 555.53 | 0 - 614.0 | 11.79 - 890.62 |
|  | A | A | A | A | AB | B | AB | AB |
| **G3** | 45.36 | 26.92 | 20.54 | 28.93 | 21.85 | 24.75 | 26.21 | 93.11 |
|  | 0 - 126.77 | 0 - 107.99 | 14.11 - 75.32 | 6.73 - 354.64 | 12.66 - 584.04 | 7.60 - 321.00 | 7.32 - 138.00 | 8.77 - 302.85 |
|  | A | A | A | A | AB | AB | AB | AB |
| **G4** | 35.71 | 10.79 | 42.21 | 283.87 | 20.91 | 37.44 | 93.00 | 125.66 |
|  | 0 - 120.92 | 0 - 69.72 | 8.46 - 107.99 | 6.01 - 489.35 | 5.43 - 47.71 | 8.98 - 747.56 | 2.78 - 680.49 | 28.57 - 719.01 |
|  | A | A | A | A | AB | AB | AB | AB |

The letters show the differences between the intergroup comparisons.
